# Quantifying the Link between Anatomical Connectivity, Gray Matter Volume and Regional Cerebral Blood Flow: An Integrative MRI Study

**DOI:** 10.1371/journal.pone.0014801

**Published:** 2011-04-15

**Authors:** Bálint Várkuti, Mustafa Cavusoglu, Alexander Kullik, Björn Schiffler, Ralf Veit, Özge Yilmaz, Wolfgang Rosenstiel, Christoph Braun, Kamil Uludag, Niels Birbaumer, Ranganatha Sitaram

**Affiliations:** 1 Institute of Medical Psychology and Behavioral Neurobiology, University of Tübingen, Tübingen, Baden-Württemberg, Germany; 2 Max Planck Institute for Biological Cybernetics, Tübingen, Baden-Württemberg, Germany; 3 Graduate Training Centre of Neuroscience, International Max Planck Research School, Tübingen, Baden-Württemberg, Germany; 4 Wilhelm-Schickard Institute for Computer Engineering, University of Tübingen, Tübingen, Baden-Württemberg, Germany; 5 Institute of Psychology, Albert-Ludwigs-University, Freiburg im Breisgau, Baden-Württemberg, Germany; 6 CIMeC - Centro interdipartimentale Mente/Cervello, Rovereto, Trento, Italy; 7 Ospedale San Camillo, Istituto di Ricovero e Cura a Carattere Scientifico, Venezia, Veneto, Italy; Indiana University, United States of America

## Abstract

**Background:**

In the graph theoretical analysis of anatomical brain connectivity, the white matter connections between regions of the brain are identified and serve as basis for the assessment of regional connectivity profiles, for example, to locate the hubs of the brain. But regions of the brain can be characterised further with respect to their gray matter volume or resting state perfusion. Local anatomical connectivity, gray matter volume and perfusion are traits of each brain region that are likely to be interdependent, however, particular patterns of systematic covariation have not yet been identified.

**Methodology/Principal Findings:**

We quantified the covariation of these traits by conducting an integrative MRI study on 23 subjects, utilising a combination of Diffusion Tensor Imaging, Arterial Spin Labeling and anatomical imaging. Based on our hypothesis that local connectivity, gray matter volume and perfusion are linked, we correlated these measures and particularly isolated the covariation of connectivity and perfusion by statistically controlling for gray matter volume. We found significant levels of covariation on the group- and regionwise level, particularly in regions of the Default Brain Mode Network.

**Conclusions/Significance:**

Connectivity and perfusion are systematically linked throughout a number of brain regions, thus we discuss these results as a starting point for further research on the role of homology in the formation of functional connectivity networks and on how structure/function relationships can manifest in the form of such trait interdependency.

## Introduction

Recent findings are beginning to shed light on the organisational principles behind the structure of the brain [1]. Instead of studying the brain merely as an agglomeration of individual regions with their very specific functions and structural idiosyncrasies, this research, with its new and more systemic perspective, is trying to understand the fundamental lines along which structure/function relationships form [2]–[5].

Such principles are identified by studying the parts (e.g. brain regions) and analysing the global properties of the entire system that emerge from links between the parts (e.g. white matter connections). The network of white matter connections in the brain seems to adhere to a small-world organisation principle, defined by short path lengths for reaching any part from any other part, while providing high clustering and highly efficient wiring.

Once such a property is established, relations to properties of other systems can be analysed.

The shared small-world properties of interregional gray matter structural similarity [6]–[7] and white matter connectivity, and the small-world properties of functional brain networks as assessed with electroencephalography [8], are recent examples that certain common principles of organisation can be found in a multitude of brain systems and on a number of scales [9].

As some of these traits, such as number of synapses, cell body population, perfusion and type and quantity of neural fiber bundles have proven to be examinable using advanced imaging methods of spectroscopy, perfusion, structural or diffusion MRI, brain regions can now be characterised regarding multiple traits at once. In certain cases the relationship between different structural properties is formed in a systematic way, e.g. larger brain regions tend to have more connections than smaller brain regions. In other cases, structural and functional traits are also systematically coupled, e.g. the neural computations that take place in a particular brain region are partially shaped by the quantity, quality and usage of the in- and outgoing connections of that region. In turn the principle of functional (computational) segregation - as reflected in the typology of unimodal motor and sensory or heteromodal association areas of the cortex - is known to be mirrored both in the macroscopic white matter network topology [10] and in functional connectivity networks [11]. The afferent and efferent white matter connections of the motor system, the ascending pathways of the primary sensory cortices and the rich interconnectivity of the association areas are not uncoupled from the function of these areas, but rather allow us to formulate hypotheses on their functional roles.

Mounting evidence suggests [12]–[15] that certain functional traits - such as the activity profile of a brain region arising from its function and its metabolic demand as reflected e.g. by local capillary density - are closely coupled [16], [17]. Local metabolic demand and perfusion are directly linked in the healthy brain, thus allowing the indirect assessment of metabolism through means of perfusion weighted imaging.

If local white matter connectivity shapes neurocomputational processes, and these processes influence local function and therefore metabolic demand, one could hypothesise that local white matter connectivity and local perfusion might be coupled throughout the brain as well. If this were the case, such coupling would constitute the manifestation of a supply-and-demand-principle - the metabolic demand being shaped by connectivity - in the formation of a previously undocumented structure/function relationship.

In order to quantify the outlined traits of perfusion and white matter connectivity we conducted an integrative MRI study on 23 healthy subjects (divided into two groups of 11 and 12 participants), utilising a combination of Diffusion Tensor Imaging (DTI), Arterial Spin Labeling (ASL) and anatomical imaging.

DTI and ASL are methods to non-invasively characterise white matter structure and gray matter function of the brain, respectively [18]–[19]. While DTI allows for the estimation of anatomical connectivity between regions of the brain, ASL represents a MRI method for the quantification of global and regional Cerebral Blood Flow (rCBF).

DTI based tractography can be used to characterise the amount and integrity of white matter tracks between two regions and allows for an estimation of connection probability. For this purpose, probability density maps can be formed from the repeated propagation of curves through the DTI-based tensor field, which is representing local white matter orientation. Currently, DTI data are integrated into graph representations [20], [21] of the white matter network in order to analyse the relation of network topology to function [22] and its impairment [23]. This type of network modelling has originated from the broader discipline of graph theory [24], which is dedicated to the understanding of the emergence of certain global and local properties of a given system from the distribution of pairwise relations of parts of that system (e.g. if many nodes have one connection to another single node, the sum of these connections or edges make that node the hub of the system).

This approach allows for the quantification of node specific traits (e.g. the number of edges connecting one node to others nodes, termed degree), edge specific traits (e.g. how severely a network is affected by the removal of an edge) and general graph properties (e.g. how efficient is the information transfer from any point A to any point B in general).

ASL [25], [26] on the other hand has proven to be a sensitive and reliable method for the quantification of gray matter perfusion, defined as the volume of arterial blood delivered to the capillary bed per unit volume of brain tissue per unit time. It has been utilised to study brain function following neuronal activation, as well as for the detection of changes of perfusion occurring during brain pathology, maturation and aging [27], [28]. This is done by assessing the inflow of magnetically labelled arterial water spins into an imaging slice. For quantitative measurement of rCBF, ASL constructs images following a tagging of inflowing arterial blood by a 180° radiofrequency inversion pulse and, in an interleaved fashion, acquiring control images without prior tagging, so that the subtraction of these two images (control-tag) only leaves magnetisation proportional to the blood flow.

So far, only limited attention has been paid to possible synergies from the combined use of these two imaging modalities. As a result, the link between white matter network connectivity and rCBF has not yet been systematically addressed. Currently, there are a few studies focusing on rCBF alterations and changes in white matter integrity (e.g. level of myelinisation, orientation of fibers) as markers of Alzheimer's disease (AD) and mild cognitive impairment [29], but the number of systematic studies using state of the art imaging of both modalities in the same sample is low. White matter lesions in the frontal lobe were found [30] to be correlated with a lower CBF in the elderly. [31] reported that rCBF reductions in the parietal cortex are correlated with white matter integrity reductions in the Posterior Cingulum in a healthy sample. However, evidence for a systematic relationship between white matter network topology and rCBF has yet to be established.

Following our hypothesis that local metabolic demands are largely determined by the connectivity profile of a brain region, we assumed that local perfusion and local connectivity measures of gray matter regions are to be correlated. In order to take into account the properties of the gray matter regions themselves (e.g. a region with larger volume might show higher perfusion and higher degree), we integrated a Voxel-based Morphometry (VBM) analysis into our design. VBM is an established method [32], [33] for the quantification of volumetric differences for the entire brain and its subparts based on anatomical MRI images.

Our approach allows for the analysis of the entire trait triplet of gray matter volume (GMV), white matter connectivity and perfusion at once.

**Figure 1:**
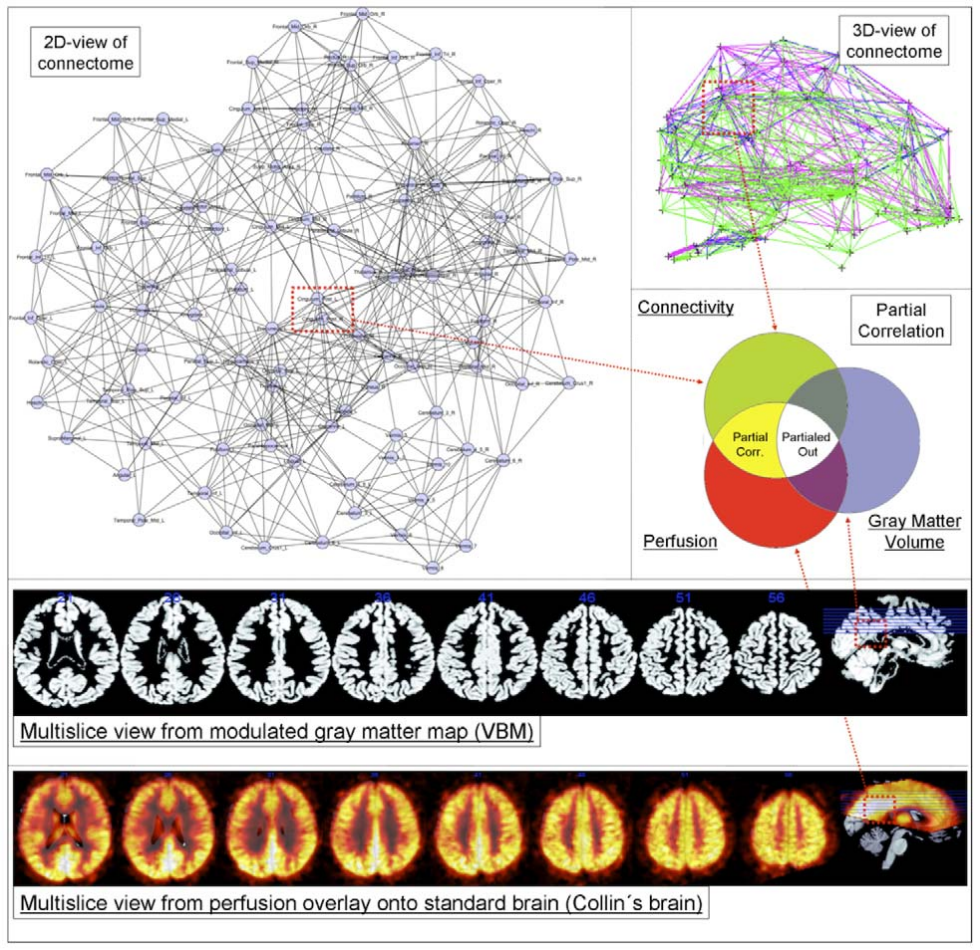
General principle. Graph theoretical properties of the nodes (in this example Posterior Cingulate) are correlated with their relative perfusion (multi-slice example in low row) while controlling for GMV (multi-slice example in upper row) - left-upper 2D graph in background formed by using the Spring Embedder Algorithm on a group averaged connectome for cortico-cortical sparsity 11%. In our PC approach we partial out the statistical influence of GMV in order to assess the covariation of perfusion and connectivity directly.

Using this multi-facetted data we analysed the connectivity/perfusion-covariation profiles for regions of the Automated Anatomical Labeling (AAL) atlas, while controlling for GMV (see Figure 1).

For the further analysis we divided the regions into the region classes cortical, subcortical and regions of the Default Brain Mode Network (DMN). The rationale for this division are manifold, on the one hand the graph theoretical and perfusion specific characterisation of subcortical regions is a novelty of this study, on the other hand it stands to reason that vasculature and connectivity systematically differ between cortical and subcortical regions and thus should be considered separately. Because we were particularly interested in the covariation profiles of regions with documented high resting state activity, we subdivided the class of cortical regions further into a DMN regions class. These region classes were comprehensively analysed on the individual-, group- and regionwise levels while controlling for local GMV. Furthermore we report on the correlation between perfusion with GMV, as well as GMV and perfusion with graph theoretical properties of white matter connectivity, respectively.

**Figure 2:**
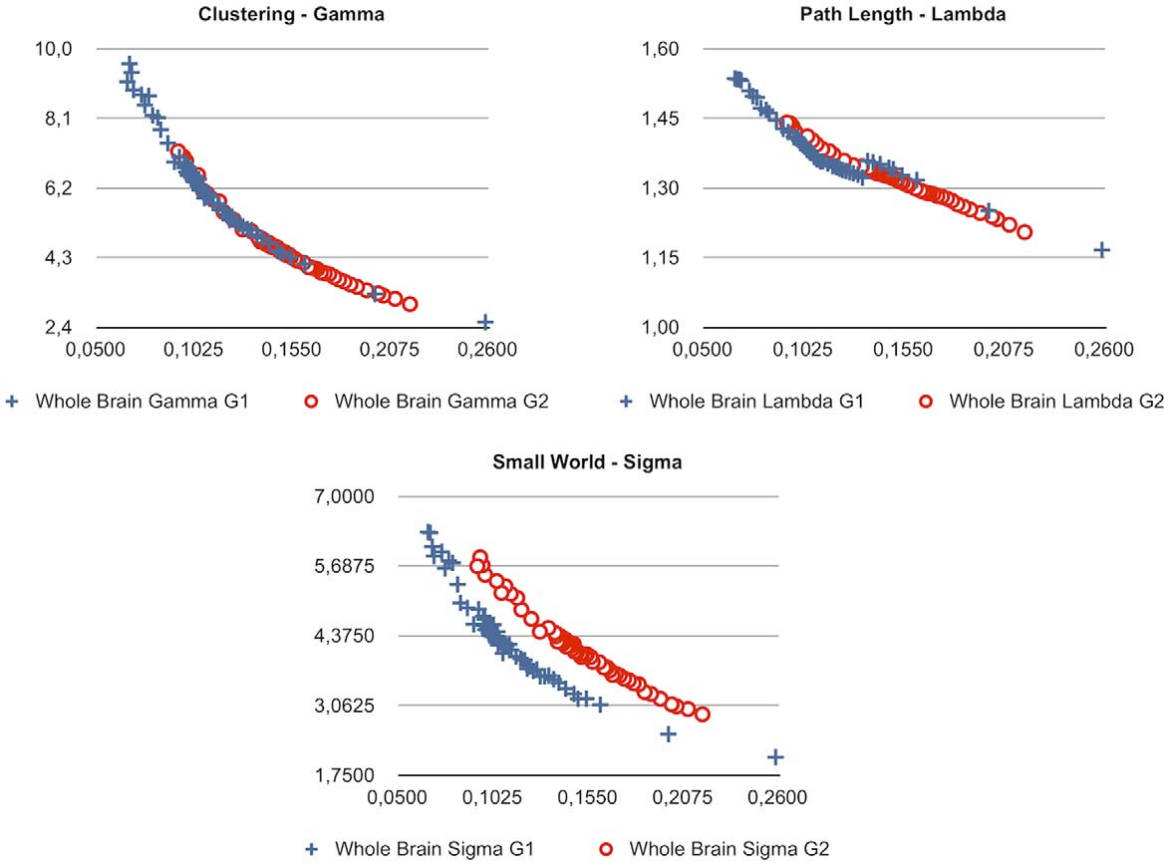
Changes in white matter topology for the whole-connectome values of Gamma, Lambda and Sigma (y-axis) and cortico-cortical sparsity (x-axis, 1 = 100%) for Group 1 and Group 2.

We employed a probabilistic tractography approach for the estimation of our white matter connectivity graphs and provide results from not one, but many plausible white matter networks (connectomes). Whereas the state of the art does currently not serve with unambiguous properties of a commonly accepted connectome standard, we provide results from a sweep over plausible edge probability thresholds and resulting cortico-cortical sparsity values in Figure 2.

## Results

### 2.1 White matter network topology

Adjacency graphs such as connectomes can be described regarding their small-world properties using path length, clustering and the ratio of both. Relative path length is calculated as the current path length in a given adjacency graph relative to the path length in an equal random graph and expressed in the coefficient Lambda. The coefficient Gamma is calculated analogously for clustering and the coefficient Sigma is defined as Gamma/Lambda ratio, respectively.

The estimated connectomes of all subjects showed small-world characteristics (Group 1, Lambda M = 1.3413 SD = 0.0121; Gamma M = 5.1155 SD = 0.5179; Sigma M = 3.6525 SD = 0.3181; Group 2, Lambda M = 1.3283 SD = 0.0218; Gamma M = 4.6459 SD = 0.54341; Sigma M = 4.2216 SD = 0.3260) as described by [10] for the plausible cortico-cortical sparsity sweep range of 11–17%.

The different DTI scanning parameters (higher number of diffusion directions and better spatial resolution for Group 2) for both groups resulted in Group 2 showing higher Sigma values for the sweep range than Group 1 for comparable sparsity values. Further it is to note that thresholding with equal edge probability values in both groups consistently lead to a higher number of accepted edges for the connectomes of Group 2.

Small-world property analyses for interregional GMV and perfusion correlations are provided in Text S1.

### 2.2 General sample characteristics

To address relation between the total rCBF in non-cerebellar regions of the brain and whole-brain graph theoretical metrics, we tested for such correlations in each group. For this analysis, whole-brain connection density, Lambda, Gamma, average degree, clustering coefficient and global efficiency values for the brain of each subject were paired with the same individual's average rCBF values (ASL measured in resting state), derived from of all non-cerebellar brain regions. These variables were tested for potential correlations (Spearman's rho) with graph metrics resulting from each step of the sweep over plausible edge probability thresholds. For each group p-values for all thresholding steps were False Discovery Rate (FDR, p<0.05) corrected. No significant results were obtained.

With respect to regional properties, as expected relative perfusion (after within-subject normalisation) was found to be higher in DMN regions than in other regions of the cortex (two-sample t-test, p = 0.0019), in accordance with our hypothesis. The difference between total perfusion (unnormalised rCBF) in regions of the DMN and the rest of the cortex showed a tendency towards significance (two-sample t-test, p = 0.0723). Regions of the DMN had significantly higher relative GMV than other regions of the cortex (two-sample t-test, p = 7.6283*10^−8^).

### 2.3 Correlation between perfusion, connectivity and GMV

**Figure 3:**
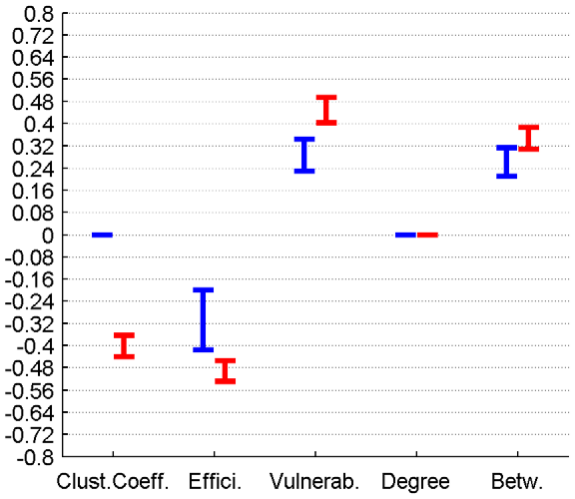
Correlation between graph theoretical metrics and perfusion for Group 1 (in blue) and Group 2 (in red), for the subcortical region class. The minima and maxima of correlation magnitudes are provided for FDR (p<0.05) corrected significant correlations (Spearman's rho), calculated with graph metrics from connectomes resulting from the sweep over plausible cortico-cortical sparsity levels (11–17% range).

**Figure 4:**
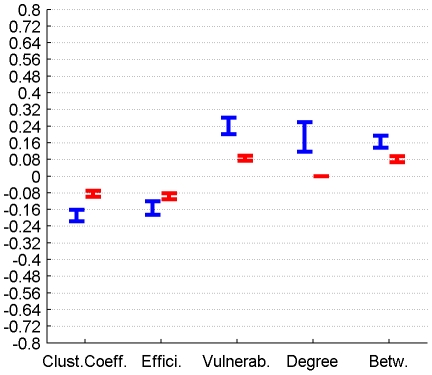
Correlation between graph theoretical metrics and perfusion for Group 1 (in blue) and Group 2 (in red), for the cortical region class. The minima and maxima of correlation magnitudes are provided for FDR (p<0.05) corrected significant correlations (Spearman's rho), calculated with graph metrics from connectomes resulting from the sweep over plausible cortico-cortical sparsity levels (11–17% range).

**Figure 5:**
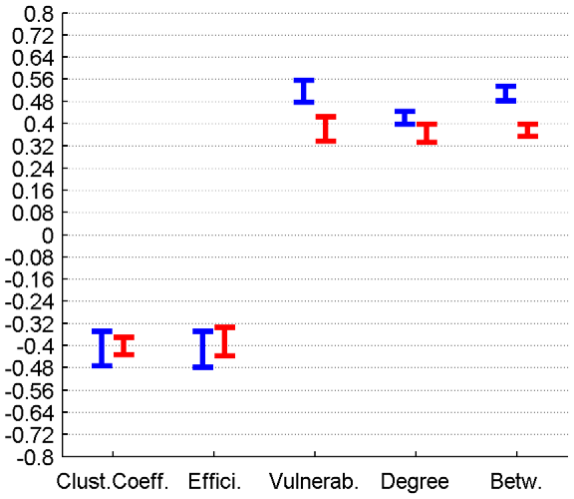
Correlation between graph theoretical metrics and perfusion for Group 1 (in blue) and Group 2 (in red), for the DMN region class. The minima and maxima of correlation magnitudes are provided for FDR (p<0.05) corrected significant correlations (Spearman's rho), calculated with graph metrics from connectomes resulting from the sweep over plausible cortico-cortical sparsity levels (11–17% range).

The correlation between perfusion and graph metrics for cortical, subcortical and DMN regions for connectomes from the plausible cortico-cortical sparsity range of 11–17% [20] with their respective minima and maxima magnitudes (Spearman's rho correlation, all FDR, p<0.05 corrected) are reported for both groups in Figures 3, 4, 5 and Table 1.

**Table 1:** Correlation between graph theoretical metrics and perfusion for Group 1 and Group 2, for the region classes subcortical, cortical and DMN.

Correlation of graph theoretical metrics and perfusion	Group		Clustering Coefficient	Efficiency	Vulnerability	Degree	Betweenness
Subcortical Regions	1	min Value	n.s.	-0,42	0,23	n.s.	0,21
Subcortical Regions	1	max Value	n.s.	-0,20	0,34	n.s.	0,31
Subcortical Regions	2	min Value	-0,44	-0,53	0,40	n.s.	0,31
Subcortical Regions	2	max Value	-0,36	-0,46	0,49	n.s.	0,39
Cortical Regions	1	min Value	-0,22	-0,19	0,20	0,12	0,14
Cortical Regions	1	max Value	-0,16	-0,12	0,28	0,26	0,19
Cortical Regions	2	min Value	-0,10	-0,11	0,07	n.s.	0,07
Cortical Regions	2	max Value	-0,07	-0,08	0,10	n.s.	0,09
DMN Regions	1	min Value	-0,48	-0,48	0,48	0,40	0,48
DMN Regions	1	max Value	-0,35	-0,35	0,56	0,44	0,53
DMN Regions	2	min Value	-0,43	-0,44	0,34	0,33	0,35
DMN Regions	2	max Value	-0,37	-0,33	0,42	0,40	0,40

The minima and maxima of correlation magnitudes are provided for FDR (p<0.05) corrected significant correlations (Spearman's rho), calculated with graph metrics from connectomes resulting from the sweep over plausible cortico-cortical sparsity levels (11–17% range).

**Figure 6:**
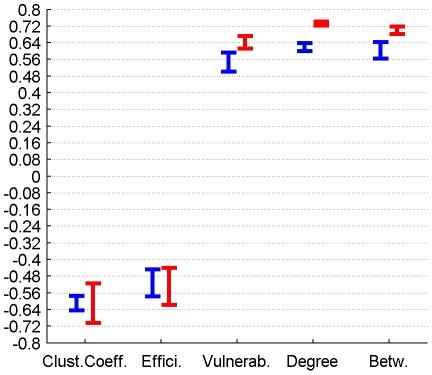
Correlation between graph theoretical metrics and GMV for Group 1 (in blue) and Group 2 (in red), for the subcortical region class. The minima and maxima of correlation magnitudes are provided for FDR (p<0.05) corrected significant correlations (Spearman's rho), calculated with graph metrics from connectomes resulting from the sweep over plausible cortico-cortical sparsity levels (11–17% range).

**Figure 7:**
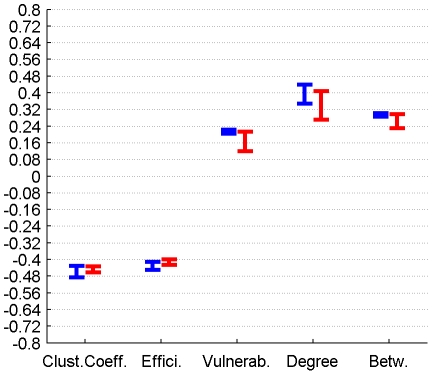
Correlation between graph theoretical metrics and GMV for Group 1 (in blue) and Group 2 (in red), for the cortical region class. The minima and maxima of correlation magnitudes are provided for FDR (p<0.05) corrected significant correlations (Spearman's rho), calculated with graph metrics from connectomes resulting from the sweep over plausible cortico-cortical sparsity levels (11–17% range).

**Figure 8:**
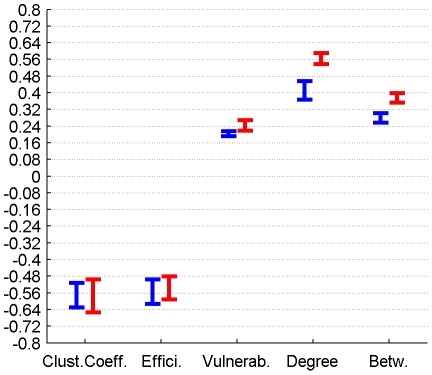
Correlation between graph theoretical metrics and GMV for Group 1 (in blue) and Group 2 (in red), for the DMN region class. The minima and maxima of correlation magnitudes are provided for FDR (p<0.05) corrected significant correlations (Spearman's rho), calculated with graph metrics from connectomes resulting from the sweep over plausible cortico-cortical sparsity levels (11–17% range).

The correlation between GMV and graph metrics for cortical, subcortical and DMN regions for connectomes from the plausible cortico-cortical sparsity range of 11–17% [20] with their respective minima and maxima magnitudes (Spearman's rho correlation, all FDR, p<0.05 corrected) are reported for both groups in Figures 6, 7, 8 and Table 2.

**Table 2:** Correlation between graph theoretical metrics and GMV for Group 1 and Group 2, for the region classes subcortical, cortical and DMN.

Correlation of graph theoretical metrics and GMV	Group		Clustering Coefficient	Efficiency	Vulnerability	Degree	Betweenness
Subcortical Regions	1	min Value	-0,64	-0,58	0,50	0,60	0,56
Subcortical Regions	1	max Value	-0,57	-0,45	0,59	0,64	0,64
Subcortical Regions	2	min Value	-0,70	-0,62	0,61	0,72	0,68
Subcortical Regions	2	max Value	-0,52	-0,44	0,67	0,74	0,72
Cortical Regions	1	min Value	-0,49	-0,45	0,20	0,35	0,29
Cortical Regions	1	max Value	-0,43	-0,41	0,22	0,44	0,30
Cortical Regions	2	min Value	-0,46	-0,43	0,12	0,27	0,23
Cortical Regions	2	max Value	-0,43	-0,40	0,21	0,41	0,30
DMN Regions	1	min Value	-0,63	-0,61	0,19	0,37	0,26
DMN Regions	1	max Value	-0,51	-0,50	0,22	0,46	0,30
DMN Regions	2	min Value	-0,65	-0,59	0,22	0,54	0,35
DMN Regions	2	max Value	-0,50	-0,48	0,27	0,59	0,40

The minima and maxima of correlation magnitudes are provided for FDR (p<0.05) corrected significant correlations (Spearman's rho), calculated with graph metrics from connectomes resulting from the sweep over plausible cortico-cortical sparsity levels (11–17% range).

**Figure 9:**
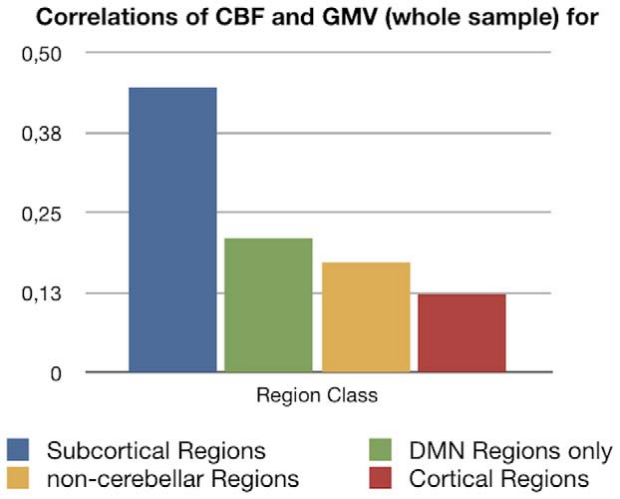
Correlation between GMV and perfusion for the whole-sample level are provided for the region classes cortical, subcortical, all non-cerebellar and DMN, all results FDR (p<0.05) corrected.

The correlation between GMV and perfusion for cortical, subcortical, all non-cerebellar and DMN regions (Pearson's correlation, all FDR, p<0.05 corrected) are reported for a whole-sample analysis (perfusion and anatomical image acquisition were similar in both groups, therefore a whole-sample analysis can be conducted exclusively for this trait pair) in Figure 9 and Table 3.

**Table 3:** Correlation between GMV and perfusion for the whole-sample level are provided for the region classes cortical, subcortical, all non-cerebellar and DMN, all results FDR (p<0.05) corrected.

Correlations of CBF and Gray Matter Volume (whole sample) for	p-value	r
Subcortical	1E-12	0,45
DBM	0,00042	0,21
non-cerebellar	5E-15	0,17
Cortical	2E-07	0,12

Both GMV and perfusion are systematically correlated with graph metrics, hence we repeated our analysis using GMV as a control variable.

### 2.4 Partial correlation between perfusion and connectivity controlling for GMV

#### 2.4.1 Group Level - Cortical Nodes

For the 78 cortical nodes the partial correlations (PCs) of perfusion and the graph theoretical metrics local clustering coefficient, local efficiency, local vulnerability, degree and betweenness (control variable: local GMV) fail to reach significance (FDR correction applied for each metric and group separately, p<0.05) in both groups for all connectome estimations from the cortico-cortical sparsity range 11–17%. Significant PCs are found for Group 1 in the form of a negative covariation of the clustering coefficient (in range 13–16%, rho between -0.095 and −0.077) and a positive covariation of vulnerability (in range 10–16%, rho between 0.144 and 0.23), degree (in range 13.5–16%, rho between 0.093 and 0.148) and betweenness (in range 11.5–16%, rho between 0.074 and 0.11) with perfusion, but are minor in magnitude. Group 2 only shows a minor covariation of perfusion and degree (in range 11.5–13.5%, rho between −0.129 and −0.09).

#### 2.4.2 Group Level - Subcortical Nodes

For the 10 subcortical nodes the PCs of perfusion and the graph theoretical metrics namely local clustering coefficient and betweenness (control variable: local GMV) fail to reach significance (FDR correction applied for each metric and group separately, p<0.05) in both groups for all connectome estimations from the cortico-cortical sparsity range 11–17%.

Significant PCs are found for both groups in the sparsity range of 13–16% in the form of a negative covariation of local efficiency with perfusion (Group 1, rho between −0.351 and −0.249, Group 2 rho between −0.3940 and −0.2979), and a positive covariation of vulnerability and perfusion (in range 12–13%, Group 1 rho between 0.2276 and 0.257, Group 2 rho between 0.1858 and 0.3012).

When groups are analysed separately, significant PCs are found for Group 2 in the form of a negative covariation of degree (in range 11.8–17%, rho between −0.2523 and −0.1932) with perfusion, but are not supported by results from Group 1.

#### 2.4.3 Group Level - DMN Nodes

In order to specifically characterise regions (such as Medial Prefrontal Gyrus, Medial Temporal Lobe and Pole, Posterior Cingulate Cortex, Precuneus, Inferior Parietal Lobe) which are associated with the Default Brain Mode [34] we singled these cortical nodes out and repeated the groupwise analysis.

**Figure 10:**
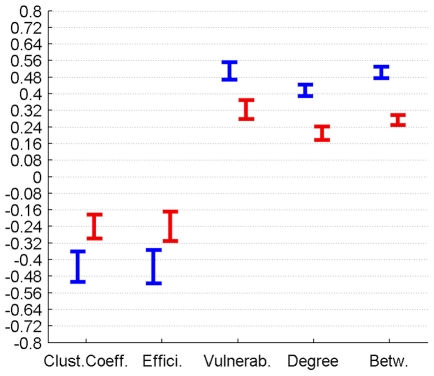
PC of graph theoretical metrics and perfusion for Group 1 (in blue) and Group 2 (in red) for the region class DMN are provided with the minima and maxima of correlation magnitudes for FDR (p<0.05) corrected significant correlations (Spearman's rho), calculated with graph metrics from connectomes resulting from the sweep over plausible cortico-cortical sparsity levels (11–17% range).

For these 12 cortical nodes the PCs of perfusion and the graph theoretical metrics local clustering coefficient, local efficiency, vulnerability, degree and betweenness (control variable: local GMV) reached significance (FDR correction applied for each metric and group separately, p<0.05) in both groups for all connectome estimations from the cortico-cortical sparsity range 11–17%. The results are summarised in Figure 10 and Table 4.

**Table 4:** PC of graph theoretical metrics and perfusion for Group 1 and Group 2 for the region class DMN are provided with the minima and maxima of correlation magnitudes for FDR (p<0.05) corrected significant correlations (Spearman's rho), calculated with graph metrics from connectomes resulting from the sweep over plausible cortico-cortical sparsity levels (11–17% range).

Partial Correlation (Spearman's Rho) with Perfusion in		Clustering Coefficient	Efficiency	Vulnerability	Degree	Betweenness
Group 1	min Value	-0,51	-0,52	0,47	0,39	0,47
Group 1	max Value	-0,36	-0,35	0,55	0,44	0,53
Group 2	min Value	-0,30	-0,31	0,28	0,18	0,25
Group 2	max Value	-0,18	-0,17	0,37	0,24	0,30

#### 2.4.4 Individual Level

For each group, double FDR correction (p<0.05) was applied separately for the PC analyses carried out for each single subject and each single edge threshold (for the plausible sparsity range). On the individual level only five subjects showed any significant covariation (four from Group 1).

The significant correlations on the individual level were all positive and minor to medium in magnitude, found only for the graph metrics degree, betweenness and vulnerability and only for nodes from the DMN and cortical regions.

#### 2.4.5 Regionwise Level

**Figure 11:**
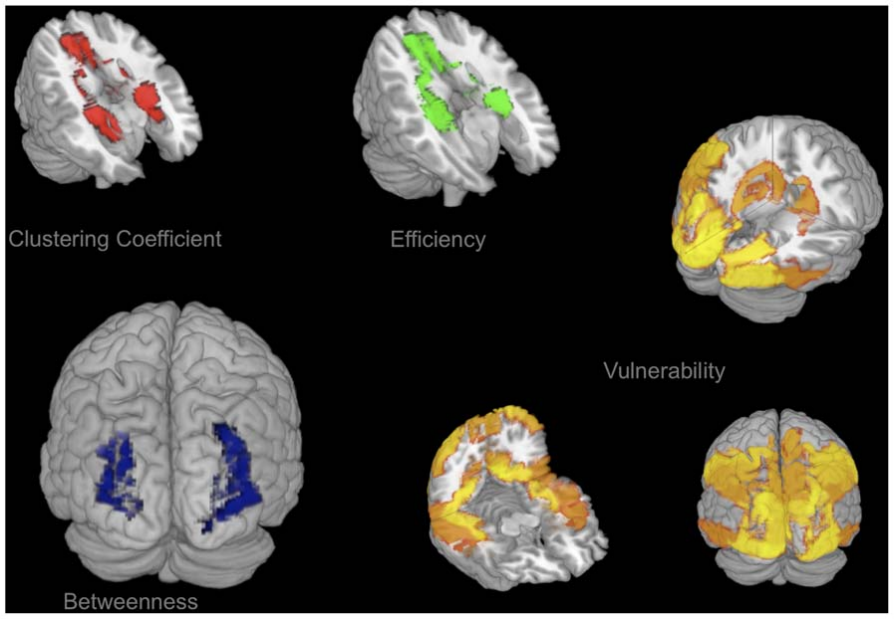
PC of graph theoretical metrics and perfusion for Group 1 and Group 2 for regionwise FDR (p<0.05). Corrected significant non-contradictory correlations (Spearman's rho) are visualised with colour intensity based on the absolute maxima of correlation magnitudes; PCs calculated with graph metrics from connectomes resulting from the sweep over plausible cortico-cortical sparsity levels (11–17% range). PC of local clustering coefficient and perfusion is negative (red, upper left), PC of local efficiency and perfusion is negative (green, upper middle), PC of local betweenness and perfusion is positive (blue, lower left), PC of local vulnerability and perfusion is positive (yellow, lower right).

The perfusion, GMV and five graph theoretical metric trait measures of each non-cerebellar brain region of the AAL atlas were collected from each subject into two groupwise tables, one seven-value vector per person for each edge probability threshold. As a result each region can be characterised with respect to the covariation profile of perfusion and graph theoretical anatomical connectivity metrics while controlling for local GMV. For each group, double FDR correction (p<0.05) was applied separately for the PC analyses carried out for each single region and each single edge threshold. Only covariation profiles of regions are reported that passed the double FDR correction in both groups for more than half of the edge thresholds in the plausible cortico-cortical sparsity range. The statistically significant regional covariation profiles of the five graph theoretical metrics with local perfusion are provided in the supporting information files (Table S1) with a visualisation in Figure 11.

## Discussion

To the best of our knowledge, this study represents the first attempt to quantify the relation of local perfusion and local anatomical connectivity, using the described MR sequences in combination with a probabilistic estimation of the connectivity graph. Our results on the robust small-world properties of the white matter connectomes are in accordance with previous findings.

### 3.1 Connectome topology

When conducting a sweep over the range of plausible edge probability thresholds, the properties of the white matter networks from both groups of participants show marked differences. For identical edge probability thresholds the sparsity of the resulting connectomes from Group 2 (DTI measured with a higher number of diffusion directions and a higher spatial resolution) is higher than the sparsity of connectomes in Group 1. On the other hand small-world property (Sigma) values are numerically higher for the same sparsity levels in Group 2.

It can be assumed that more edges of the white matter network are reliably identified with the superior DTI measurement parameters used in Group 2, and that the entirety of these reliably identifiable edges tend to display small-world properties. The numerically higher path length ratio values (Lambda) in Group 1 might indicate that long-distance white matter connections are measured more reliably with the superior DTI measurement parameters, thus facilitating their probabilistic tracking and acceptance as edge in the subsequent edge probability thresholding process. The acceptance of only a few more long-distance edges can decrease path length drastically, while influencing the sparsity value only minimally.

The deviating Sigma values for equal cortico-cortical sparsity in the two groups might indicate that two qualitatively different white matter networks are compared with one another. But as we presently provide whole-connectome Sigma over cortico-cortical sparsity the deviation in small-world properties may as well be attributable to superior reliability in measuring non-cortico-cortical edges for the DTI sequences applied to Group 2.

As can be seen in Figure 2 a sudden rise in path length Lambda can be observed in Group 1 between 13.3 and 13.6% cortico-cortical sparsity. A rise in path length occurring when more edges are added is unusual and indicates that a new cluster of nodes was connected to the rest of the connectome with the additional edges passing a decreasing threshold (leading to higher sparsity level). As clusters unconnected to the rest of the connectome are unlikely, this might be an indication that the true level of cortico-cortical sparsity might be in a range above 13.6%.

### 3.2 General sample characteristics

The lack of any significant relation of whole-brain graph metrics and average perfusion values indicates that there is no relation of overall absolute perfusion and overall connectivity in our healthy sample.

### 3.3 Correlation between perfusion, connectivity and GMV

Both local GMV and local perfusion show a pattern of significant covariation with local graph theoretical metrics in both groups and for all three described region classes. This pattern of covariation indicates that higher perfusion and higher GMV are more likely to be found in hub-like regions of the brain, with high degree, betweenness and vulnerability, thus with low clustering coefficient and local efficiency.

GMV and perfusion show a marked covariation profile on the whole-sample level, with the strongest correlation for subcortical regions, followed by regions of the DMN and finally non-cerebellar regions in general and cortical regions. The marked correlations of perfusion and GMV could theoretically be a confound due the fact that relatively larger regions contain more large vessels and have a superior ASL Signal-to-Noise ratio (SNR) when compared with smaller regions. Alternatively the correlations could indicate a genuine association of average perfusion and relative GMV.

### 3.4 Covariation of perfusion and connectivity

Cortical and subcortical nodes were characterised with respect to their graph theoretical properties, which were further correlated with measures of perfusion, while controlling for local GMV.

#### 3.4.1 Group Level

The covariations found on a group level, analysing the trait correlations of all cortical nodes, were only minor (<0.25) in magnitude and inconclusive in terms of cross-group stability. The covariation profile of traits from subcortical nodes on the other hand shows a negative covariation of local efficiency and a positive covariation of vulnerability with perfusion in a minor to medium (<0.6) range of magnitude.

For regions associated with the DMN we observe similar patterns of covariation, but complemented by a positive covariation of degree and betweenness and a negative covariation of the clustering coefficient with perfusion, in a range of minor to medium magnitude.

For all three classes of nodes, the magnitude of covariation observed in Group 1 exceeds the magnitude of covariations in Group 2.

#### 3.4.2 Individual Level

On the individual level correlations for all cortical, subcortical and DMN regions largely fail to reach significance in a stable pattern.

#### 3.4.3 Regionwise Level

On a regionwise level, frontal, cingular and hippocampal regions show a negative covariation of perfusion with the clustering coefficient and local efficiency, while mainly posterior portions of the brain show a positive covariation particularly with vulnerability. All covariations remain minor to medium in magnitude and have the same sign in both groups, except for the Frontal Inferior Cortex (pars triangularis and orbitalis), the Frontal Superior Medial Gyrus, the Supplementary Motor Area, the Olfactory Cortex and the Rolandic Operculum, where the signs of covariation are contradictory.

### 3.5 General covariation pattern

The present results do not allow for a clear and global falsification of a perfusion/connectivity-covariation in the brain. Rather the results on group- and regionwise levels point towards a positive covariation of rCBF with degree, betweenness and vulnerability and a negative covariation of rCBF with the clustering coefficient and efficiency for some particular regions of the brain.

The correlation results for the covariation of GMV, perfusion and connectivity (no statistical control of GMV) point into the same direction, thus could be influenced by the issues indicated in the methods section.

This might indicate that for some areas of the brain an increased rCBF is more likely to be found in regions, which have a central position in the white matter network and possess hub-like properties, but have poor local clustering and number of parallel pathways to any other node (local efficiency). This might be the manifestation of a structural organisation principle, which strives to minimise the potential of metabolic deficiencies in central nodes, which are part of a rather sequential connection architecture.

The failure of the correlations to reach significance on the individual level could be attributed to the low statistical power, since for the DMN twelve and for the subcortical regions merely ten pairs of values are correlated for each participant, further decreasing the degrees of freedom by using PC and using conservative multiple comparison correction. The same holds for results on the regionwise level (the number of value pairs correlated for each region is equal to the number of subjects in that group, which are eleven and twelve, respectively), which might offer one explanation for the contradictory findings in some regions.

The most stable results are obtained for regions of the DMN on a group- and regionwise level. Regions of the DMN are known to possess hub-like properties in terms of both anatomical and functional connectivity [4]. Possibly the present results point towards an exclusive realisation of an observable linear supply-and-demand-principle of perfusion and connectivity in these regions, although it can not be excluded that regions show possibly non-linear relations, which we did not test for in this present study.

### 3.6 Implications

These results might have implications on our understanding of resting state networks in general, not only those exclusively involving nodes of the DMN network [34]–[39].

The finding that DMN regions (e.g. Posterior Cingulate) might play a role as bottlenecks in a potentially task-negative default mode of macroscopic neural traffic might be underlined by past [34] [40] and present results on the coincidence of heightened resting state perfusion and hub characteristics (marked by high degree and vulnerability) in DMN regions. Just like the hubs of any traffic network can form bottlenecks where traffic might dam up, some nodes with a central position within the white matter network might show heightened activity during distinct global states, such as rest. But whether the apparent functional connectivity of these hubs can be attributed to genuine joint information processing in a functionally relevant network or merely to similar activity arising from e.g. coincidentally relaying traversing neural signals, or parts of both, can currently not be conclusively answered.

It is possible that the common traits (high perfusion, hub-like connectivity, large relative GMV) and the strong covariation of these traits contribute either causally to the formation, or confoundingly to our awareness of resting state networks. Similar Blood Oxygen Level Dependent (BOLD) activity profiles might in principle be caused by node interactivity in a functionally relevant information processing network or alternatively by mere similarity of isolated neural processes in individual nodes [3]. It is unclear, to what extent the coincidence of high resting state perfusion and hub-like connectivity can distort estimations of functional connectivity between such nodes, by influencing the BOLD signal from these nodes generally with their mutually high perfusion and the underlying activity in these nodes with their common hub-like connectivity.

Understanding the link between local function and local connectivity better, might help understand the apparent link between functional and anatomical connectivity networks [4]–[5] and resolve the role of mere homology in our perception of resting state networks.

It is a further open question of interest, whether there is any causal neurobiological link between a regional perfusion profile and the emergence of hub characteristics or vice versa.

### 3.7 Conclusion

The method described might hold potential for the diagnosis of various diseases, as the identified dependency of perfusion and connectivity might reflect the balance resulting from organisational principles inherent to the structural architecture of the healthy brain. Although the identified coefficients from the present healthy sample are only minor to medium in magnitude, it is to note that two traits might be only rudimentarily related in the healthy brain, but might show stronger links in the pathologically altered brain (e.g. hypoperfusion preceding gray matter atrophy).

However, to better understand the relevance of the identified relationships and to explore changes of the derived coefficients due to ageing or disease future studies have to be performed on the respective populations.

### 3.8 Potential confounds

It can be assumed that during our resting state ASL measurement, the regions of the DMN showed their - by definition - heightened resting state activity and metabolic demand and it is possible that our measurements were therefore systematically biased. If the metabolic profile of a region is best assessed when this region shows a stable amount of activity over time, the metabolic demand of the resting state active brain regions (the DMN) was eventually captured more clearly by our resting state experiment, than the metabolic demand profile of a less resting state involved region such as e.g. the Fusiform Gyrus. This issue touches on the general question whether stimulus-free measurements constitute a reliable baseline measurement for the whole brain, or only for regions active under stimulus-free conditions.

Thus it can not be excluded that covariations of the present form could be identified more conclusively for others regions of the brain as well. For such experiments, these regions would need to be consistently activated for a prolonged amount of time (e.g. by presentation of a series of faces for characterisation of the Fusiform Gyrus), what might allow us to assess their metabolic demand during activity better.

It might be argued that the prospect of such studies might be limited, as brain regions do not merely show a straightforward type of homogeneous functional specialisation, but are rather able to perform heterogenous functions that depend on the task-specific network(s) they are integrated in at a point of time. Nevertheless it should be possible to capture the range of metabolic demand spanned by the conditions of activity and rest for each region of the brain, as any computational process is likely to influence metabolic demand in a specific way.

Studies on the relation of functional and anatomical connectivity networks in general might be influenced by a similar aspect. The definition of functional connectivity is always limited to the state of the brain the connectivity is observed in, therefore the definition of network nodes is not state-independent (the stable association of the nodes with a network is one but not the only defining trait of the nodes) and thus probably not one-by-one mappable onto other data, e.g. to adequately assess the underlying anatomical connectivity network.

It stands to reason that both the range of metabolic demand states, local connectivity and GMV, and the probability ranking of affiliations to functional connectivity networks of a brain region might be related in some manner to the shape of the Hemodynamic Response Function in that region.

Alternatively to a direct causal interpretation, the co-variation of perfusion and connectivity could as well be caused by a confounding third, presently unregarded variable other than GMV. Our initial hypothesis on the link between local connectivity and local neural computations, neural computations and neural activity, and neural activity and metabolic demand involves a series of dependencies, making the influence of intermediary variables highly likely.

A more comprehensive model, including more potentially intermediate variables beyond merely GMV, could be used in future studies to investigate the relations of local computational processes (e.g. using electroencephalographic methods), neural circuitry (e.g. by utilising information on local cell body types), neurotransmitter concentrations (e.g. by using Magnetic Resonance Spectroscopy), metabolism and connectivity at once. With such a larger and more comprehensive dataset it is likely that the particular perfusion/connectivity balance profile of each region could be resolved further.

### 3.9 Methodological issues

Several methodological issues need to be addressed. Although we employed a probabilistic tracking approach with a high number of random walks and conservative thresholding, presently it is not possible to ensure the validity of one singular estimated connectome with complete certainty due to the methodological difficulty of proper edge falsification and the strong dependency of the results upon the chosen brain parcellation scheme (the AAL atlas in this case). For a more precise estimation, the methodology has to be refined by extending it to more sophisticated graph estimation methods e.g. based on Q-ball imaging data [41] or diffusion tensor studies utilising multi b-value imaging - and by combining these advanced measurements with advanced brain function atlases, derived from large databases of functional connectivity data, since the optimal anatomical connectivity network nodes stem from the parcellation of the brain into those regions, which eventually form a multitude of functional cooperations with others but always do this as a whole [4].

The combination of multiple methods (DTI based connectivity estimation, VBM, ASL) allows for quantifying and describing brain regions with a high number of measures, both on a global as well as on a regional scale. The proper interpretation of this multifaceted data, with respect to the multiple comparisons problem and open questions on the combination of non-gaussian measures with classical statistical approaches, needs to be advanced in an integrative manner.

Further as the present findings are based on cross-sectional data from small samples, the results could be influenced by potential cohort or small sample size effects. A longitudinal study design with a higher number of subjects is necessary to broaden our understanding of the link of these structural and functional brain properties.

It is to note that many different methods with their individual assumptions and errors are combined in an integrative study, and that any of the modules (probabilistic tractography, graph theoretical analysis, anatomical image segmentation, perfusion measurement, artefact control, normalisation etc.) could clearly be improved on their own. Our approach is merely intended as a starting point for the combination of the provided measures.

Therefore the present findings are of a preliminary nature, as for the integration of information about local perfusion, connectivity and gray matter properties, the issues of natural variability versus the distribution of measurement error have to be further resolved. Other quantification approaches for measuring integrity or impairment of gray matter such as spectroscopy of synaptic density or recently introduced Positron Emission Tomography methods [42] focussing on the quantification of plaques and tangles in AD might complement the present approach in the future [4].

## Methods

### 4.1 Subjects and data acquisition

Eleven participants (5 females) were recruited for Group 1 from a student sample (average age = 25.4 years, SD = 3.4 years, Range: 21–32). After an update of scanner software twelve further participants (7 females) were recruited, referred to as Group 2 (average age = 36.7 years, SD = 10.7 years, Range: 23–57). Participants of both groups gave written informed consent. All participants were right handed (as confirmed by the Edinburgh Handedness Questionnaire) and both physically (confirmed by extensive health questionnaire) and mentally healthy (confirmed by the german ICD-10-Symptomrating questionnaire). The experiments were approved by the local ethics committee. All MR data were acquired on a 3T Siemens MAGNETOM Trio TIM (Erlangen, Germany) scanner using a 12-channel head coil. The head of each subject was bedded in a deflatable pillow so as to minimise head motion artefacts.

### 4.2 Anatomical data acquisition and processing

Anatomical images were acquired using a T1 weighted sequence using a 3D MP-RAGE (magnetisation prepared - rapid acquisition gradient echo) sequence (1×1×1 mm voxels, TR = 7.92, TE = 2.48, Flip Angle = 16°, FoV = 256*256, 192 transversal slices, Group 1 - 1×1×1.1 mm voxels, TR = 2.3, TE = 2.98, Flip Angle = 9°, FoV = 230*256, 160 sagittal slices, Group 2). The brain was extracted from the raw image using the robust iterative estimation function (Fractional intensity threshold = 0.5) of the Brain Extraction Tool and subsequently segmented into gray and white matter ([43], distributed within the FMRIB's Software Toolbox - FSL 4.0; http://www.fmrib.ox.ac.uk/fsl).

### 4.3 DTI data acquisition

Each subject in Group 1 participated in a DTI measurement (1.3×2.4×2.4 mm voxels, no gap, TR = 8.83 sec, TE = 98 ms, FoV = 1360*1360, Flip Angle = 90°, 50 transversal slices, 12 diffusion directions, two averages, b-value = 1000 s/mm^2^) with the field of view (FoV) comprising the full cerebrum and parts of the rostral cerebellum like the Uvula and Tuber of Vermis, Flocculus and Crus Cerebelli (dependent on individual overall brain size).

Each subject in Group 2 participated in a DTI measurement (1.8×1.8×2.2 mm voxels, no gap, TR = 6.8 sec, TE = 93 ms, FoV = 1782*1840, Flip Angle = 90°, 50 transversal slices, 64 diffusion directions, two averages, b-value = 1000 s/mm^2^) with the FoV comprising the full cerebrum and parts of the rostral cerebellum. The DTI data were processed using the DTI and Fibertools Software Package [44] as described in the section Network Edge Definition.

### 4.4 Connectome construction and edge calculation

For an overview on data flow for each participant and employed analysis schemes please see Figure S1 and Figure S2.

#### 4.4.1 Network node definition

In order to define the network nodes, gray matter areas were labelled for each subject individually based on the AAL atlas [45], resulting in 116 nodes (80 cortical, 10 subcortical, 26 cerebellar) by using the procedure of normalisation and parameter inversion analogous to the method described in [20].

In order to transfer the images into DTI native space, T1 weighted structural images were coregistered with the B0 (non-diffusion) image and then normalised to the Montreal Neurological Institute (MNI) space. The resulting transformation was inverted to warp the AAL template from MNI space to the DTI space. The discrete labeling values were preserved by using a nearest neighbour interpolation method. Normalisation and inverse transformation were implemented using the SPM8 package.

All available subcortical (Caudate, Putamen, Pallidum, Hippocampus and Thalamus) and cerebellar areas, which were within the individual DTI FoV were included as nodes in order to round out the validity of the individual connectivity graph estimation.

#### 4.4.2 Network edge tracking

Using in-house code, the white matter voxels, which were neighbouring the gray matter of each network node, were defined as seed voxels of that area.

Only voxels with a Fractional Anisotropy (FA) value above 0.3 (gray matter FA can reach values up to ∼0.2, [46]) were admitted to this procedure. In addition, these voxels also had to be labelled as white matter by the segmentation step and reside within the brain outline mask resulting from the iterative Brain Extraction Step.

Probabilistic tracking from the seed points was realised by using the PiCo [47] approach. The number of random walks was adjusted to the number of voxels within the white matter tracking area for each single seed point.

The algorithms implemented in the DTI and Fibertools Software Package [44] allow for creating extended visitation maps for the tracking from each seed set (all seed voxels of an AAL area) separately, based on the curves originating from the seed points and being propagated through the tensor field (number of curves equalling the number of random walks) and combine this information with statistical estimates on the plausibility of confluence of two white matter tracts anywhere in the brain. Please see [44] for more precise information, as our definition of edge probability is based on the Probability Index of forming a part of the Bundle of Interest (PIBI) value concept developed by [44].

Although theoretically one single edge probability value can be chosen for edge-thresholding based on estimates of the cortico-cortical white matter network sparsity (the percentage of accepted edges, relative to the amount of possible edges) derived from the literature, we provide our results in the form of a sweep over a range of plausible thresholds - in order to avoid false conclusions originating in the limitations of only one threshold-specific white matter network (see [48]).

In order to substantiate this sweep, to ensure the validity of the present findings and to avoid false conclusions due to erroneous selection of the edge probability threshold, we performed a number of connectome estimations based on a large range of thresholds (step width 2.5*10^−9^). The applied thresholds ranged from implausibly low thresholds (average edge probability in non-zero voxels min. 10^−10^) allowing for a very high number of accepted edges to overly conservative high thresholds (average non-zero edge probability min. 8*10^−7^) making the adjacency matrix very sparse respectively. The selection of the threshold for calculation of the graph metrics employed in the present work was based on both empirical and theoretical considerations and reproduces values for cortico-cortical sparsity, clustering, average path length and resulting small-world coefficients, which are comparable with the results described by [20]. For better comparison the figures are provided with our measure of sparsity of cortico-cortical edges only, resulting from the applied thresholds. Changes of overall graph metrics over various thresholds (decreasing small-world coefficient Sigma with decreasing threshold, resulting from higher Gamma values and constant Lambda) are as well in concordance with the literature.

It is to be noted that a less conservative threshold, allowing for a higher number of edges within each individuals’ connectome seems to result in increasing PCs of vulnerability (and other metrics) with perfusion for both cortical and subcortical network nodes. This might indicate that the real relation is even stronger than depicted in this context, since there is no other overt reason why the consideration of implausible edges with lower connection probability (which appear only when the threshold is decreased) should systematically increase the PCs. Instead the presented results of our sweep over possible thresholds show that reforming the white matter network topology by adding more edges systematically strengthens statistical relations of perfusion and connectivity.

Further, as for the same threshold more edges are identified for the superior DTI measurement scheme (please see Figure S3), this could mean that equal sparsity for both groups can only be attained by using a more conservative threshold for Group 2. The deviating Sigma for identical cortico-cortical sparsity in Group 2 indicates that either other cortico-cortical or additional non-cortico-cortical edges can be found with higher statistical plausibility, when data from advanced DTI measurements is utilised. The fact that with a higher statistical plausibility higher Sigma can be observed for identical sparsity further validates the small-world property as an inherently stable feature of the white matter connectome.

Particularly interesting is the fact that edge probability shows a power-law style distribution (as well for far lower thresholds, data not shown), indicating that for a low number of edges probability values are very high, while for a very large number of possible edges probability values are very low. How this is related to the observed power-law distribution of hubs for certain edge probability thresholds could be subject of further numerical simulations.

#### 4.4.3 Definition of Graph metrics

**Figure 12:**
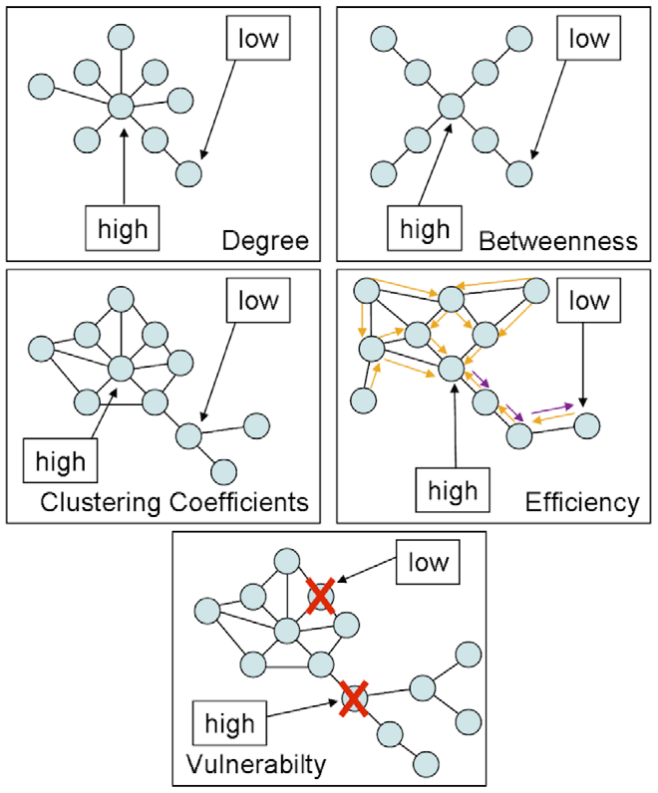
Illustration of utilised node-level graph metrics.

For each node, edge and resulting overall graph the available metrics were calculated using the scripts provided within the Brain Connectivity Toolbox [49]. The vulnerability metric was calculated as described by [20]. Please see Figure 12 for illustration of the different graph metrics.

It is to be noted, that graph analysis of white matter connectivity has the advantage that connectivity is not restricted to concepts of direct links only, which is often the case in classical Region-of-Interest to Region-of-Interest deterministic tractography, but incorporates notions of indirect (path length >1) connectivity in all utilised graph metrics but degree.

**Degree** is the number of edges connecting one node by a path length of one with other nodes (e.g. a hub has a high degree vs. an isolated node with only one connection to the rest of the network has a low degree). In order to normalise local degree values to make them comparable across graphs, the values are divided by the sum of all node degree values.

A node with high **betweenness** is at a central position of the network, meaning that many of the paths connecting any node A and any node B traverse that node (e.g. while a central hub has high betweenness, a node forming a cul-de-sac has the lowest betweenness). Betweenness is calculated as the fraction of all shortest paths in the network, which traverse a given edge/node. In order to normalise local betweenness values to make them comparable across graphs, the values are divided by the sum of all node betweenness values within the graph.

If the nearest neighbours (path length  =  1) of a node are also directly connected to each other they form a cluster (e.g. when an individual's friends are also friends with one another). The **clustering coefficient** quantifies the number of connections that exist between the nearest neighbours of a node as a proportion of the maximum number of possible connections amongst them. This normalisation allows comparing the clustering coefficient of two nodes directly, irrespective of their individual degree.

**Efficiency** is inversely related to minimum path length (shortest) and quantifies how easily one node can be reached from any other node. Nodes with a high efficiency have many interconnected neighbours and are thus more easily reachable via a number of (parallel) direct paths from any other node. Local efficiency of a node is therefore calculated as the harmonic mean for neighbour-neighbour distances.

The **vulnerability** metric describes how strongly the average shortest path lengths (the mean distance to get from any node A to any node B) in a network grow if a node is removed. If a node with high betweenness, high degree, low efficiency and low clustering coefficient is removed, a central hub is eliminated leading to insufficient cost-effective (short) detours for reconnecting severed nodes and high node vulnerability. If a network is fully connected (all nodes are connected to all other nodes) degree, efficiency and clustering coefficient might be high for all nodes, but vulnerability might be low, as the removal of one node would not affect the average distance of any pair of nodes significantly, due to the high number of parallel connecting paths/cost-effective detours.

### 4.5 ASL data acquisition

ASL data were acquired with a FAIR-QUIPSSII PASL encoding scheme with echo-planar imaging (EPI) readout. A total of 201 alternating tag and control images were obtained in each run (total scan time 10 min, TI1 = 700 ms, TI2 = 1100 ms, TE = 20 ms, TR = 3000 ms, voxel size = 3.5×3.5×10 mm). For ASL measurements of Group 2 the number of slices was increased from ten to eleven (TI2 was changed to 1400 respectively). In order to determine the equilibrium magnetisation for absolute CBF quantification, the same parameters as above were used except that the TR and TI2 were 10000 ms and 4000 ms, respectively [50]. For analysing the ASL data, FSL software (http://www.fmrib.ox.ac.uk/fsl), self-written MATLAB (The Mathworks, Natick, MA, USA) and Linux shell script routines were used. Time courses of all voxels were motion-corrected utilising the MCFLIRT module of FSL using the mean volume of the corresponding run as reference. CBF time series were created by calculating control-tag difference images using surround subtraction (i.e., computing the difference between each image and the average of its two nearest neighbors), thereby reducing BOLD signal contamination of the CBF time course [50].

For the ASL resting state data acquisition subjects were instructed to relax with closed eyes while staying awake. The ASL values for the nodes were extracted by averaging the absolute CBF (in ml/100 g-min) from the voxels within each network node Volume of interest (VOI) for each subject.

Regional absolute and within-subject normalised rCBF values are provided for all non-cerebellar regions of the AAL atlas in the Figure S4.

### 4.6 Gray matter characterisation through Voxel-Based Morphometry

In order to address the relation between gray matter characteristics, graph theoretical measures and resting state perfusion, we performed a VBM Analysis of the T1 weighted anatomical scans so as to derive descriptives regarding the relative volume of each AAL region. As described in the section Network Node Definition, each anatomical image was skull stripped and the Voxel-based Morphometry Toolbox (VBM5.1, v.1.15, by Christian Gaser) was used subsequently for estimation of the individual modulated and unmodulated segmentation outputs. As the modulated outputs can be corrected for non-linear warping only and therefore make any further correction for different brain size redundant, these images can be used directly for volume estimations.

For the unified segmentation approach (repeated segmentation, bias correction and warping iterations as described in [51]) used in this study the tissue probability maps provided within the SPM5 template set were used since the subjects were drawn from the appropriate population. We applied the thorough clean-up option of the VBM toolbox and made use of a medium Hidden Markov Random Field model for an optimal denoising of the T1 image.

A check of sample homogeneity of the modulated images (using the standard deviation approach within VBM5.1) revealed that the VBM results of the images were all within a tolerable range.

So as to optimise the validity of our GMV estimation, we performed a voxel-wise multiplication of each modulated gray matter image with the coregistered corresponding perfusion image (containing absolute CBF information). Although perfusion imaging of white matter regions of the brain is possible in principle [52], with many sequences estimation accuracy is limited due to the longer transit delay time (the travelling time of blood from labelling region to reach the tissue) of white matter. As perfusion in gray matter is higher, such a multiplication significantly sharpens the gray matter image histogram, thus facilitating the valid estimation of GMV. In order to smooth the image histogram we applied a three dimensional Gaussian smoothing kernel (FWHM = 3 mm, being significantly below the rounded down cubic root of the volume of the smallest AAL-region in equal voxel-space). For each AAL region (network node) the number of gray matter voxels within the atlas derived volumes of interest was counted - equalling the regional volume as relative to the entire individual brain. Naturally these volume values are strongly correlated across our healthy sample as they all measure brain part volumes for identical regions.

Regional GMV values are provided for all non-cerebellar regions of the AAL atlas in Figure S5.

### 4.7 Employed data level and artefact control

As a univariate factor analysis revealed significant interindividual variability of rCBF values and an effect of gender, the analysis was performed using within-subject z-scored rCBF values. Following this approach, graph metrics and the GMV of nodes were all normalised (z-scored) on a within-subject level. The normalisation was performed separately for cortical and subcortical nodes.

Cortical or subcortical regions for which no connections could be found (due to a tracking failure) or for which no perfusion data could be obtained (due to localisation outside of the ASL imaging FoV) were excluded pairwise from the analysis (in Group 1 seven missing values in different regions, in Group 2 four missing values in two different regions). Cerebellar regions were not considered for correlation analysis, as for 30.94% of cerebellar regions rCBF could not be measured.

VOI specific artefacts in the estimation of rCBF can not be fully eliminated, due to the generally increased SNR ratio of the ASL signal and the higher chance of intravascular artefacts in larger VOIs, which naturally contain more large arteries and veins that distort rCBF estimations. Also imperfect slice profiles, remaining magnetisation transfer effects and blood tissue water exchange time are factors, which cause artefacts on rCBF estimation. In order to decrease the impact of VOI size on the estimation of relations, we included GMV estimations from VBM as a control variable into our analysis and restricted the presented results to the significant PCs calculated with Spearman's rho.

The data derived from the entirety of these measures constitute for each member of the sample a subjectwise region-by-trait table, with the columns representing local perfusion, local GMV and five graph theoretical metrics of local white matter connectivity, and with 116 rows - one for each region of the AAL atlas.

In order to interpret such multifaceted data one can take various perspectives. The data can be sorted and the trait measurements can be correlated on an individual subjectwise level, to answer the question, whether there is a significant PC - controlling for local GMV - between local perfusion and local connectivity for all the 116 regions of the AAL atlas in the brain of a given subject. Alternatively the data can be normalised on a within-subject level and integrated with data from all other subjects into a group table, to answer the question whether the previously outlined PC is significant in an analysis of pooled data as well. As a second alternative approach, the data can be sorted by region, so as to answer the question whether in pooled group data local perfusion and local connectivity of some regions shows stronger correlation than others (e.g. the left Precentral Gyrus displays a stronger PC, than the same two traits of the right Precentral Gyrus). Figures of dataflow and analaysis schemes are depicted in Figure S1 and Figure S2.

Results are presented for white matter connectomes that show plausible cortico-cortical sparsity (between 11 and 17%).

## Supporting Information

Figure S1 Dataflow of each participant is illustrated; please see Methods section for details. For each participant one table like in the lower left corner of the image results from the combination of all the measures, the node-specific graph metrics part of that table changes for each edge probability thresholding step while the rCBF and GMV parts stay constant.
(10.24 MB TIF)

Figure S2 Illustration for the group, individual and regionwise analysis schemes. C indicates the control variable GMV in the PC approach.
(10.24 MB TIF)

Figure S3 Upper: Distribution of cortico-cortical sparsity over identical edge probability thresholds for both groups. Lower: Distribution of whole-brain sparsity over identical edge probability thresholds for both groups; edge probability thresholds (x-axis) become more conservative towards the right end of the x-axis (higher threshold) leading to lower resulting sparsity due to less accepted edges.
(1.82 MB TIF)

Figure S4 Upper: Regional total perfusion in all 23 subjects (y-axis in ml/100g-min, x-axis AAL region code). Lower: Regional within-subject normalised perfusion in all 23 subjects for cortical regions (y-axis z-score, x-axis AAL region code).
(12.04 MB TIF)

Figure S5 Relative GMV of non-cerebellar Regions for all 23 subjects (y-axis number of voxels from non-linear warping only corrected modulated output image-space, x-axis AAL region code).
(10.24 MB TIF)

Table S1 Results of the regionwise PC analysis (graph theoretical metrics with perfusion, controlling for local GMV), presented for statistically significant (FDR corrected, p<0.05) regions only provided with minima and maxima of correlation magnitudes for Group 1 and Group 2.
(0.04 MB XLS)

Text S1 Notes on the small-world characteristics of binary adjacency graphs defined by the significant correlations of cortical perfusion and GMV values.
(0.04 MB PDF)

## References

[1] Bassett DS, Bullmore E (2006). Small-world brain networks.. The neuroscientist.

[2] Greicius MD, Supekar K, Menon V, Dougherty RF (2008). Resting-state functional connectivity reflects structural connectivity in the default mode network..

[3] van den Heuvel M, Mandl R, Luigjes J, Hulshoff Pol H (2008). Microstructural organization of the cingulum tract and the level of default mode functional connectivity.. Journal of Neuroscience.

[4] Buckner RL, Sepulcre J, Talukdar T, Krienen FM, Liu H (2009). Cortical hubs revealed by intrinsic functional connectivity: mapping, assessment of stability, and relation to Alzheimer's disease.. Journal of Neuroscience.

[5] Honey CJ, Sporns O, Cammoun L, Gigandet X, Thiran JP (2009). Predicting human resting-state functional connectivity from structural connectivity.. Proceedings of the National Academy of Sciences.

[6] He Y, Chen ZJ, Evans AC (2007). Small-world anatomical networks in the human brain revealed by cortical thickness from MRI.. Cerebral Cortex.

[7] Chen ZJ, He Y, Rosa-Neto P, Germann J, Evans AC (2008). Revealing modular architecture of human brain structural networks by using cortical thickness from MRI.. Cerebral cortex.

[8] Stam C, Jones B, Nolte G, Breakspear M, Scheltens P (2007). Small-World Networks and Functional Connectivity in Alzheimer's Disease.. Cerebral Cortex.

[9] Sporns O (2006). Small-world connectivity, motif composition, and complexity of fractal neuronal connections.. Biosystems.

[10] Sporns O, Zwi JD (2004). The small world of the cerebral cortex.. Neuroinformatics.

[11] Achard S, Salvador R, Whitcher B, Suckling J, Bullmore E (2006). A resilient, low-frequency, small-world human brain functional network with highly connected association cortical hubs.. Journal of Neuroscience.

[12] Kuschinsky W (1991). Coupling of function, metabolism, and blood flow in the brain.. Neurosurgical review.

[13] Farkas E, Luiten PG (2001). Cerebral microvascular pathology in aging and Alzheimer's disease.. Progress in Neurobiology.

[14] Arthurs OJ, Boniface S (2002). How well do we understand the neural origins of the fMRI BOLD signal?. TRENDS in Neurosciences.

[15] Magistretti PJ (2006). Neuron-glia metabolic coupling and plasticity.. Journal of Experimental Biology.

[16] Gjedde A, Diemer NH (1985). Double-tracer study of the fine regional blood-brain glucose transfer in the rat by computer-assisted autoradiography.. Journal of cerebral blood flow and metabolism: official journal of the International Society of Cerebral Blood Flow and Metabolism.

[17] Klein B, Kuschinsky W, Schrock H, Vetterlein F (1986). Interdependency of local capillary density, blood flow, and metabolism in rat brains.. American Journal of Physiology- Heart and Circulatory Physiology.

[18] Detre JA, Wang J, Wang Z, Rao H (2009). Arterial spin-labeled perfusion MRI in basic and clinical neuroscience.. Current opinion in neurology.

[19] Jellison BJ, Field AS, Medow J, Lazar M, Salamat MS (2004). Diffusion tensor imaging of cerebral white matter: a pictorial review of physics, fiber tract anatomy, and tumor imaging patterns.. American Journal of Neuroradiology.

[20] Gong G, He Y, Concha L, Lebel C, Gross DW (2009). Mapping anatomical connectivity patterns of human cerebral cortex using in vivo diffusion tensor imaging tractography.. Cerebral Cortex.

[21] Bullmore E, Sporns O (2009). Complex brain networks: graph theoretical analysis of structural and functional systems.. Nature Reviews Neuroscience.

[22] Li Y, Liu Y, Li J, Qin W, Li K (2009). Brain anatomical network and intelligence.. PLoS Comput Biol.

[23] Cammoun L, Gigandet X, Sporns O, Thiran JP, Maeder P (2009). Connectome alterations in schizophrenia.. NeuroImage.

[24] Barabási AL, Crandall RE (2003). Linked: The new science of networks.. American journal of Physics.

[25] Williams DS, Detre JA, Leigh JS, Koretsky AP (1992). Magnetic resonance imaging of perfusion using spin inversion of arterial water.. Proceedings of the National Academy of Sciences of the United States of America.

[26] Wong EC, Buxton RB, Frank LR (1997). Implementation of quantitative perfusion imaging techniques for functional brain mapping using pulsed arterial spin labeling.. NMR in Biomedicine.

[27] Parkes LM, Rashid W, Chard DT, Tofts PS (2004). Normal cerebral perfusion measurements using arterial spin labeling: reproducibility, stability, and age and gender effects.. Magnetic Resonance in Medicine.

[28] Biagi L, Abbruzzese A, Bianchi MC, Alsop DC, Del Guerra A (2007). Age dependence of cerebral perfusion assessed by magnetic resonance continuous arterial spin labeling.. Journal of Magnetic Resonance Imaging.

[29] Schuff N, Zhu XP (2007). Imaging of mild cognitive impairment and early dementia.. British Journal of Radiology.

[30] Kawamura J, Meyer JS, Ichijo M, Kobari M, Terayama Y (1993). Correlations of leuko-araiosis with cerebral atrophy and perfusion in elderly normal subjects and demented patients.. British Medical Journal.

[31] Jahng G, Schuff N, Du A, Zhang Y, Mueller S (2007). Age-related Reductions of Cerebral Blood Flow and White Matter Integrity by High-Field Perfusion and Diffusion MRI..

[32] Ashburner J, Friston KJ (2001). Why voxel-based morphometry should be used.. Neuroimage.

[33] Mechelli A, Price CJ, Friston KJ, Ashburner J (2005). Voxel-based morphometry of the human brain: methods and applications.. Current Medical Imaging Reviews.

[34] Raichle ME, MacLeod AM, Snyder AZ, Powers WJ, Gusnard DA (2001). A default mode of brain function.. Proceedings of the National Academy of Sciences of the United States of America.

[35] Gusnard DA, Raichle ME (2001). Searching for a baseline: functional imaging and the resting human brain.. Nature Reviews Neuroscience.

[36] Calhoun VD, Adali T, Hansen LK, Larsen J, Pekar JJ (2003). ICA of functional MRI data: an overview..

[37] Jafri MJ, Pearlson GD, Stevens M, Calhoun VD (2008). A method for functional network connectivity among spatially independent resting-state components in schizophrenia.. Neuroimage.

[38] Fair DA, Cohen AL, Dosenbach NU, Church JA, Miezin FM (2008). The maturing architecture of the brain's default network.. Proceedings of the National Academy of Sciences.

[39] Buckner RL, Vincent JL (2007). Unrest at rest: default activity and spontaneous network correlations.. Neuroimage.

[40] van den Heuvel MP, Mandl RC, Kahn RS, Hulshoff Pol HE (2009). Functionally linked resting-state networks reflect the underlying structural connectivity architecture of the human brain.. Human brain mapping.

[41] Sotiropoulos SN, Bai L, Morgan PS, Constantinescu CS, Tench CR (2010). Brain tractography using Q-ball imaging and graph theory: Improved connectivities through fibre crossings via a model-based approach.. NeuroImage.

[42] Nordberg A (2010). Amyloid Imaging in Early Detection of Alzheimer’s Disease.. Neurodegenerative Diseases.

[43] Smith SM (2002). Fast robust automated brain extraction.. Human Brain Mapping.

[44] Kreher BW, Schnell S, Mader I, Il'yasov KA, Hennig J (2008). Connecting and merging fibres: Pathway extraction by combining probability maps.. NeuroImage.

[45] Tzourio-Mazoyer N, Landeau B, Papathanassiou D, Crivello F, Etard O (2002). Automated anatomical labeling of activations in SPM using a macroscopic anatomical parcellation of the MNI MRI single-subject brain.. Neuroimage.

[46] Le Bihan D, Mangin JF, Poupon C, Clark CA, Pappata S (2001). Diffusion tensor imaging: concepts and applications.. Journal of magnetic resonance imaging.

[47] Parker GJ, Haroon HA, Wheeler-Kingshott CA (2003). A framework for a streamline-based probabilistic index of connectivity (PICo) using a structural interpretation of MRI diffusion measurements.. Journal of Magnetic Resonance Imaging.

[48] Sporns O, Tononi G, Kotter R (2005). The human connectome: a structural description of the human brain.. PLoS Comput Biol.

[49] Rubinov M, Sporns O (2009). Complex network measures of brain connectivity: uses and interpretations..

[50] Cavusoglu M, Pfeuffer J, Ugurbil K, Uludag K (2009). Comparison of pulsed arterial spin labeling encoding schemes and absolute perfusion quantification.. Magnetic resonance imaging.

[51] Ashburner J, Friston KJ (2005). Unified segmentation.. Neuroimage.

[52] van Osch MJ, Teeuwisse WM, van Walderveen MA, Hendrikse J, Kies DA (2009). Can arterial spin labeling detect white matter perfusion signal?. Magnetic Resonance in Medicine.

