# Supplementary figures and images for: Quantifying the Link between Anatomical Connectivity, Gray Matter Volume and Regional Cerebral Blood Flow: An Integrative MRI Study
Source: PLoS One. 2011 Apr 15;6(4):e14801. doi: 10.1371/journal.pone.0014801 (PMC3078126; doi:10.1371/journal.pone.0014801)

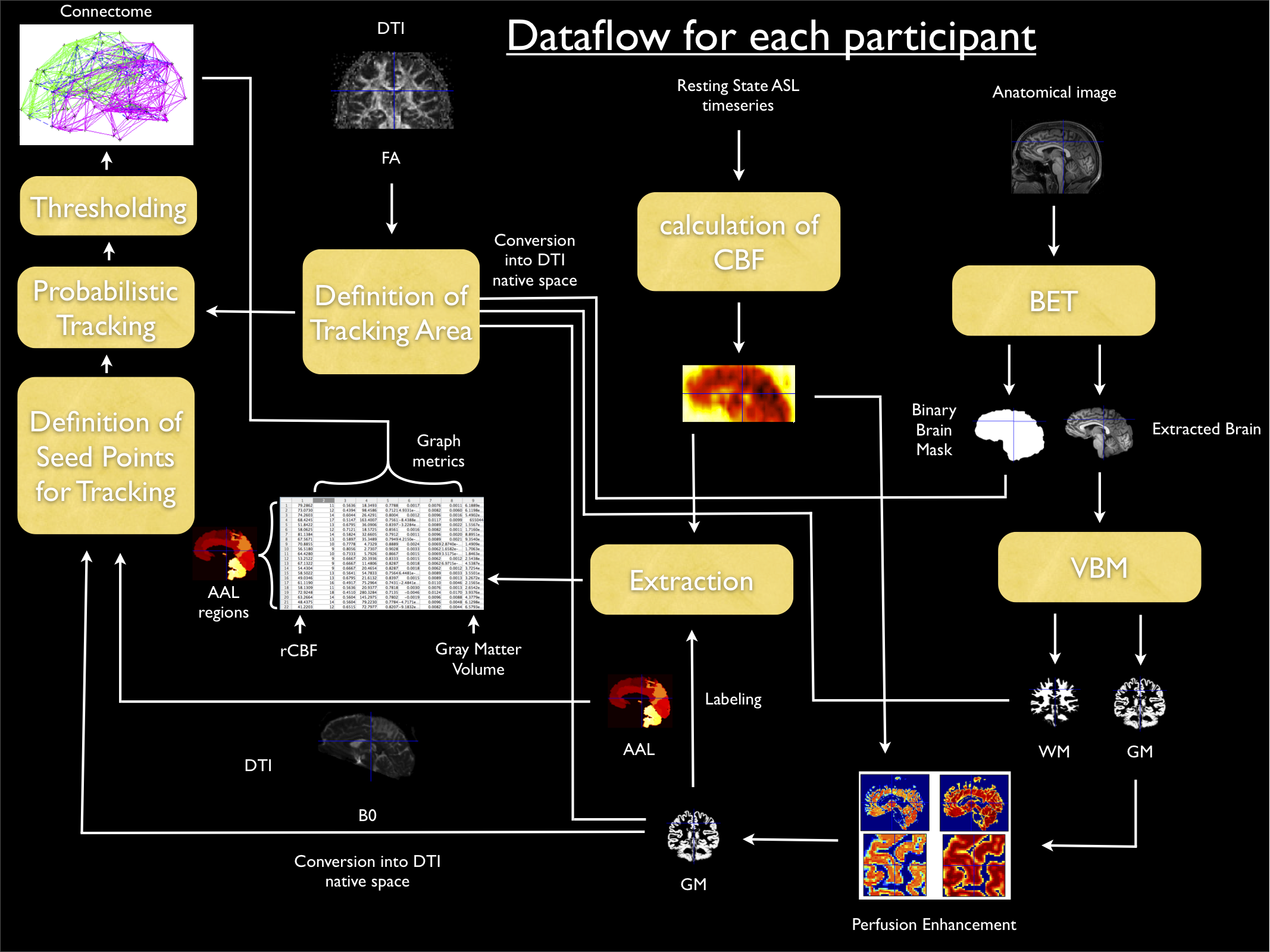

Supplement: Figure S1 — Dataflow of each participant is illustrated; please see Methods section for details. For each participant one table like in the lower left corner of the image results from the combination of all the measures, the node-specific graph metrics part of that table changes for each edge probability thresholding step while the rCBF and GMV parts stay constant. (10.24 MB TIF) [file pone.0014801.s001.tif]

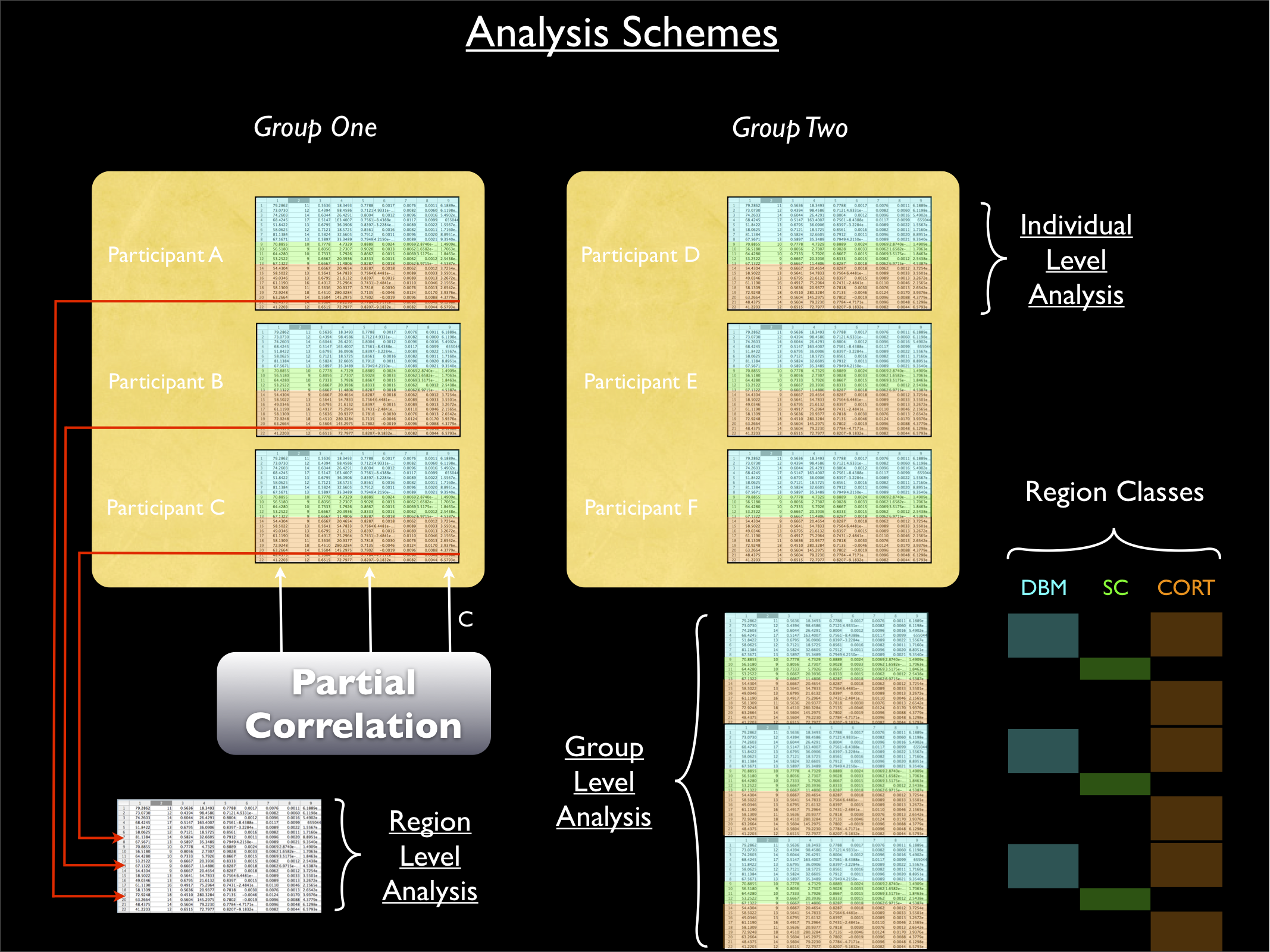

Supplement: Figure S2 — Illustration for the group, individual and regionwise analysis schemes. C indicates the control variable GMV in the PC approach. (10.24 MB TIF) [file pone.0014801.s002.tif]

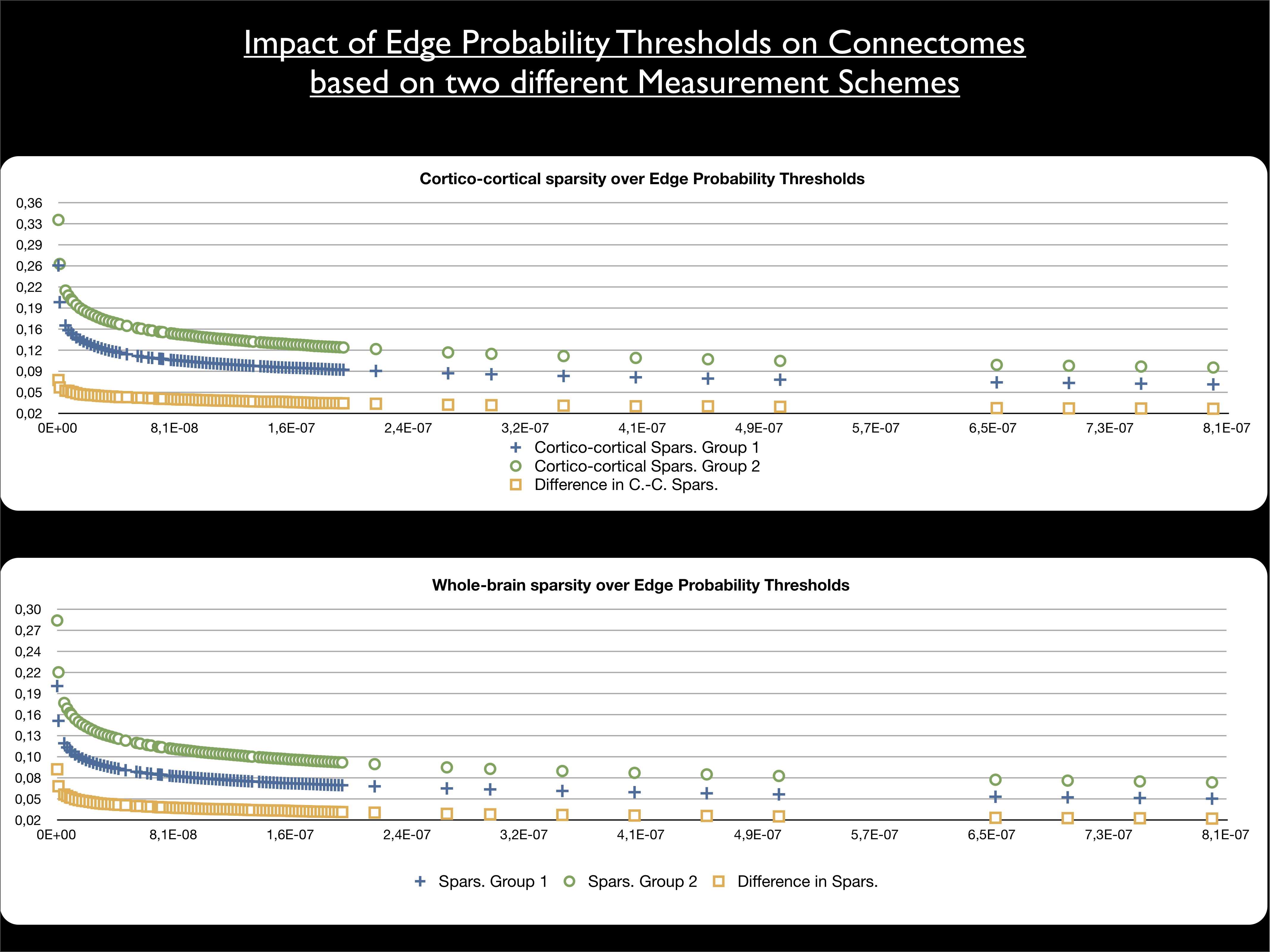

Supplement: Figure S3 — Upper: Distribution of cortico-cortical sparsity over identical edge probability thresholds for both groups. Lower: Distribution of whole-brain sparsity over identical edge probability thresholds for both groups; edge probability thresholds (x-axis) become more conservative towards the right end of the x-axis (higher threshold) leading to lower resulting sparsity due to less accepted edges. (1.82 MB TIF) [file pone.0014801.s003.tif]

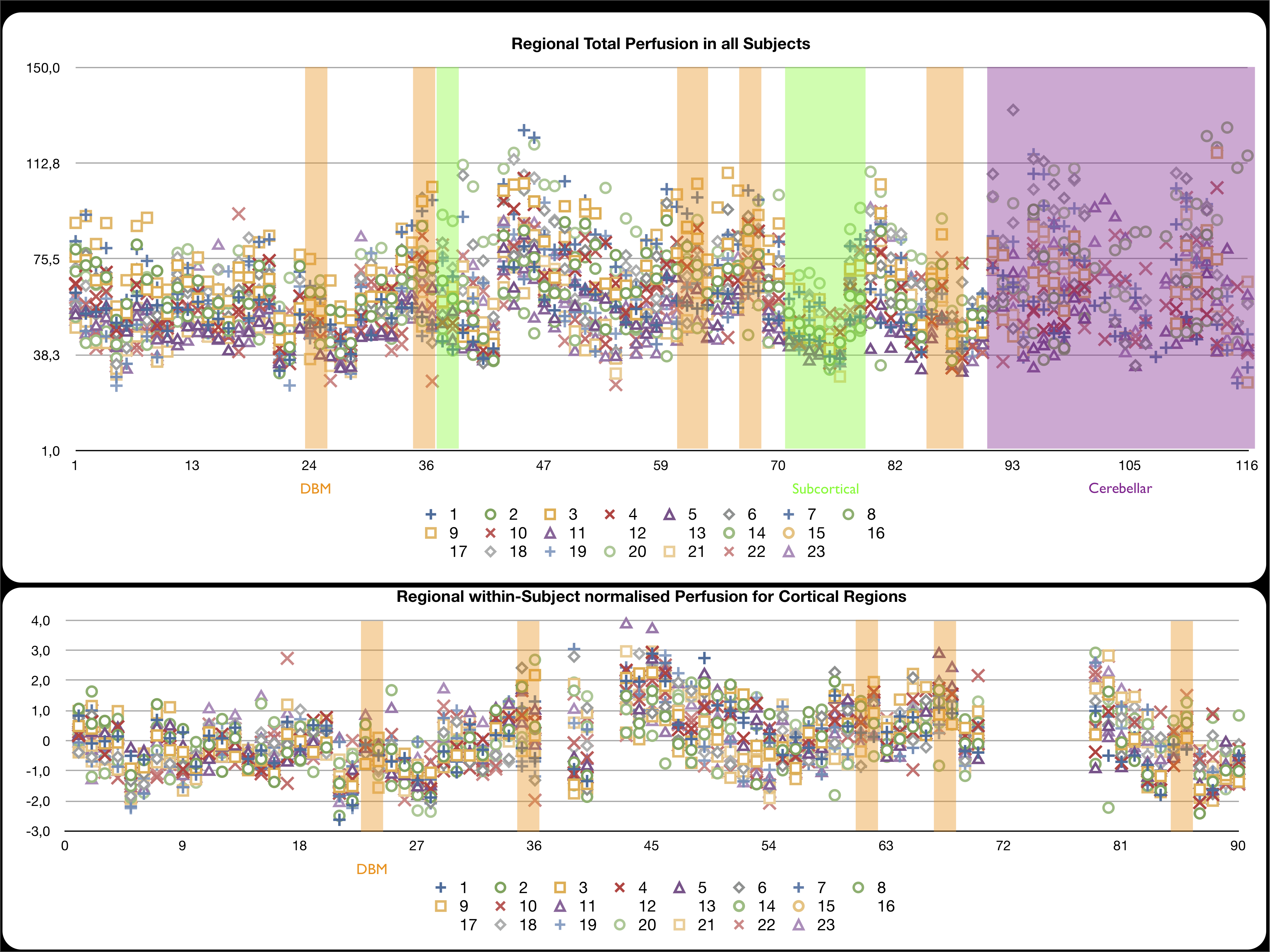

Supplement: Figure S4 — Upper: Regional total perfusion in all 23 subjects (y-axis in ml/100g-min, x-axis AAL region code). Lower: Regional within-subject normalised perfusion in all 23 subjects for cortical regions (y-axis z-score, x-axis AAL region code). (12.04 MB TIF) [file pone.0014801.s004.tif]

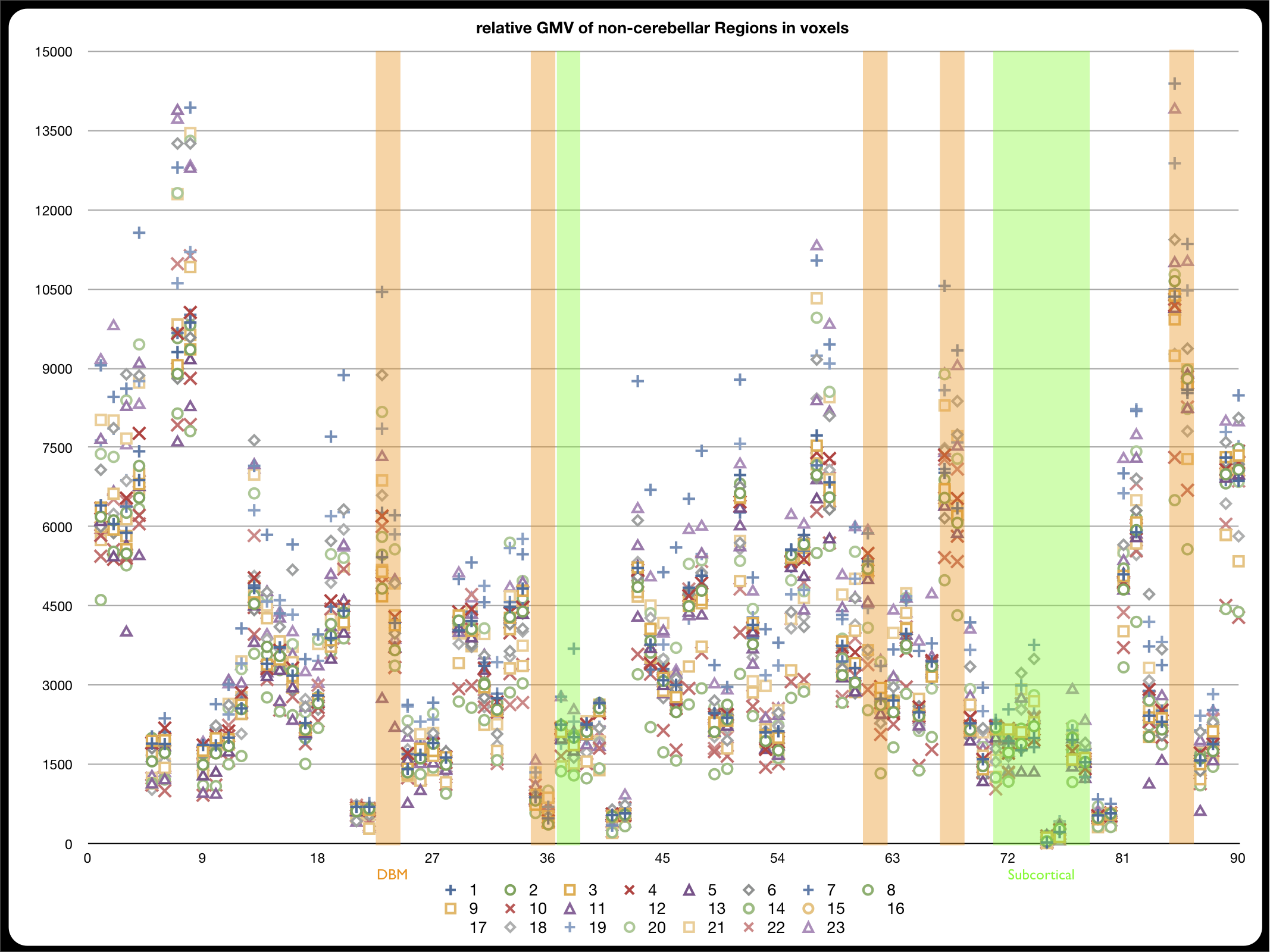

Supplement: Figure S5 — Relative GMV of non-cerebellar Regions for all 23 subjects (y-axis number of voxels from non-linear warping only corrected modulated output image-space, x-axis AAL region code). (10.24 MB TIF) [file pone.0014801.s005.tif]
