# Supplementary material for: Quantifying the Link between Anatomical Connectivity, Gray Matter Volume and Regional Cerebral Blood Flow: An Integrative MRI Study
Source: PLoS One. 2011 Apr 15;6(4):e14801. doi: 10.1371/journal.pone.0014801 (PMC3078126; doi:10.1371/journal.pone.0014801)
Supplement: Text S1 — Notes on the small-world characteristics of binary adjacency graphs defined by the significant correlations of cortical perfusion and GMV values. (0.04 MB PDF) [file pone.0014801.s007.pdf]

Supplementary Text S1

Small-World characteristics and gray matter

Following earlier findings on the organisation of cortical thickness [6,7] we analysed the small-world properties of binary adjacency graphs defined by the significant correlations (Pearson´s) of the GMVs of cortical AAL regions (n=78) for the entire sample.

As recommended by He, Chen and Evans [6] we used a False Discovery Rate (FDR) procedure for the definition of an appropriate threshold ($p<0.01$). Our results for the small-world properties (Gamma=1.1423, Lambda=1.0219, Sigma=1.3925) of regional GMVs show weaker small-world properties for regional GMV correlations than published literature findings on the correlations of regional cortical thickness.

Small-World characteristics and perfusion

By applying the same method, previously used for the gray matter analysis, but now for cortical rCBF values the binary adjacency graphs obtained by the significant interregional correlations ($p<0.01$, FDR) do show the small-world properties Gamma=1.3582, Lambda=1.0624, Sigma=1.2784 for non-normalised rCBF values and Gamma=1.0161, Lambda=1.0743, Sigma=0.9458 for within subject normalised perfusion values (relative perfusion).

The fact that interregional correlations of perfusion did not show strong small-world properties in our study, indicates that whereas small-world properties are a rather robust feature of related networks the small-world principle might not necessarily be omnipresent in every feature of the brain.
